# Glutamic Acid Residues in HIV-1 p6 Regulate Virus Budding and Membrane Association of Gag

**DOI:** 10.3390/v8040117

**Published:** 2016-04-25

**Authors:** Melanie Friedrich, Christian Setz, Friedrich Hahn, Alina Matthaei, Kirsten Fraedrich, Pia Rauch, Petra Henklein, Maximilian Traxdorf, Torgils Fossen, Ulrich Schubert

**Affiliations:** 1 Institute of Virology, Friedrich-Alexander University Erlangen-Nürnberg (FAU), Erlangen 91054, Germany; Melanie.Friedrich@viro.med.uni-erlangen.de (M.F.); Christian.Setz@viro.med.uni-erlangen.de (C.S.); Friedrich.Hahn@viro.med.uni-erlangen.de (F.H.); Alina.Matthaei@viro.med.uni-erlangen.de (A.M.); Kirsten.Fraedrich@viro.med.uni-erlangen.de (K.F.); Pia.Rauch@viro.med.uni-erlangen.de (P.R.); 2 Institute of Biochemistry, Charité Universitätsmedizin-Berlin, Berlin 10117, Germany; Petra.Henklein@charite.de; 3 Department of Otorhinolaryngology, Head and Neck Surgery, Friedrich-Alexander University Erlangen-Nürnberg (FAU), Erlangen 91054, Germany; maximilian.traxdorf@uk-erlangen.de; 4 Department of Chemistry and Center for Pharmacy, University of Bergen, Bergen N-5007, Norway; Torgils.Fossen@kj.uib.no

**Keywords:** HIV-1, Gag p6, l-domains, virus budding, membrane association, ESCRT, Tsg101, ALIX, ubiquitination

## Abstract

The HIV-1 Gag p6 protein regulates the final abscission step of nascent virions from the cell membrane by the action of its two late (l-) domains, which recruit Tsg101 and ALIX, components of the ESCRT system. Even though p6 consists of only 52 amino acids, it is encoded by one of the most polymorphic regions of the HIV-1 *gag* gene and undergoes various posttranslational modifications including sumoylation, ubiquitination, and phosphorylation. In addition, it mediates the incorporation of the HIV-1 accessory protein Vpr into budding virions. Despite its small size, p6 exhibits an unusually high charge density. In this study, we show that mutation of the conserved glutamic acids within p6 increases the membrane association of Pr55 Gag followed by enhanced polyubiquitination and MHC-I antigen presentation of Gag-derived epitopes, possibly due to prolonged exposure to membrane bound E3 ligases. The replication capacity of the total glutamic acid mutant E0A was almost completely impaired, which was accompanied by defective virus release that could not be rescued by ALIX overexpression. Altogether, our data indicate that the glutamic acids within p6 contribute to the late steps of viral replication and may contribute to the interaction of Gag with the plasma membrane.

## 1. Introduction

The HIV-1 Gag polyprotein Pr55 is necessary and sufficient for the generation of virus like particles (VLPs) [1,2]. Starting with, or shortly after virus release, Gag is processed by the viral protease (PR) into its structural components matrix (MA), capsid (CA), nucleocapsid (NC), and its C-terminal p6 protein, as well as the two spacer peptides SP1 and SP2. Upon activation of PR, the Gag components undergo structural rearrangements within the virion, leading to the formation of a cone shaped core structure, which is typical for an infectious virus particle [3]. MA directs the Gag polyprotein to the plasma membrane and lines the inner shell of the mature virion [4,5]. CA constitutes the conical core enclosing the NC, which mediates uptake and condensation of the viral genome into newly formed virions [6,7]. The C-terminal p6 region of Gag regulates the membrane fission of nascent virions from the cell surface by the action of its two late assembly (l-) domains. The primary l-domain, the PTAP tetrapeptide motif, mediates the recruitment of Tsg101 (tumor susceptibility gene 101) to the site of virus assembly [8,9,10,11]. The secondary l-domain constituted by the YPX_3_L motif regulates the binding of Gag to ALIX (ALG-2 interacting protein X) [12,13,14,15]. Although the exact contribution of ALIX to virus release has not been completely resolved yet, it can restore budding defects under conditions where binding of Tsg101 to Gag is impaired [13]. Tsg101 and ALIX are both associated to the ESCRT (endosomal sorting complex required for transport) machinery, which plays pivotal roles in membrane fission events during membrane protein trafficking and cytokinesis; processes that are topologically equivalent to virus budding [16,17,18,19,20].

The multifunctional p6 protein undergoes various post-translational modifications, including sumoylation at Lys-27 and mono-ubiquitination at Lys-27 and -33 [21,22,23]. Furthermore, it is the predominant phosphoprotein of HIV-1 particles [24]. However, the biological relevance of its multiple modifications still remains to be elucidated [22,25,26]. It has already been shown that mutation of the highly conserved PTAP motif in p6 causes a severe budding defect [27,28]. Additionally, the mutation of Ser-40 in p6 to Phe, which frequently emerges upon antiviral treatment, delays CA-SP1 processing and enhances membrane association of Gag, but causes no defect in virus release [25,29,30,31,32]. The increase in membrane association of p6 mutants correlates with an augmented entry of Gag into the UPS (ubiquitin proteasome system) and results in enhanced MHC-I antigen presentation of Gag-derived epitopes [32,33].

Even though the coding region of p6 is highly polymorphic within the HIV-1 viral genome, it contains motifs, some of which are highly conserved and enable interaction with several cellular (ERK-2, aPKC, ALIX, Tsg101, SUMO-1, ubiquitin) and viral (Vpr) proteins [8,9,10,12,21,22,26,34,35,36,37].

Alignment of group M consensus sequences revealed that p6 contains seven conserved Glu residues, which contribute to a calculated isoelectric point of 4.48, not counting the potential phosphorylation sites. In this study, we demonstrate that mutation of these Glu residues within p6 to Ala impairs Gag processing and virus release and enhances membrane association of Gag which is accompanied by increased polyubiquitination and entry of Gag into the MHC-I antigen presentation pathway.

The cumulative data indicate that these negatively charged Glu residues within p6 contribute to Gag-membrane-interaction and thus to the late functions of p6 in virus budding.

## 2. Materials and Methods

### 2.1. Cell Culture and Transfection

HeLa cells were maintained in DMEM containing 10% (*v*/*v*) inactivated fetal calf serum (FCS), 2 mM l-glutamine, 100 U/mL penicillin, and 100 μg/mL streptomycin. HeLa-K^b^ cells were cultivated in DMEM with additional 1 mg/mL geneticin. All cell culture media and reagents were purchased from Gibco (Life Technologies, Carlsbad, CA, USA).

HeLa cells were transfected with Lipofectamine 2000 (Life Technologies) according to the manufacturer’s protocol. 24 h post transfection cells were lysed in RIPA buffer (150 mM NaCl, 50 mM Tris-HCl pH 8.0, 1% NP-40, 0.5% Na-deoxycholate, 0.1% SDS, 10 mM EDTA) containing protease inhibitor cocktail cOmplete (Roche, Mannheim, Germany), 5 mM *N*-ethylmaleimide (NEM), and 1 mM phenylmethylsulfonylfluoride (PMSF).

### 2.2. Cultivation and Preparation of Primary Cells

Human tonsils were received a few hours after excision, during routine tonsillectomy, from the Department of Otorhinolaryngology, Head and Neck Surgery, Universitätsklinikum Erlangen, Germany. The cells were prepared and infected as described earlier [37,38]. Human lymphocyte aggregate cultures (HLAC) were prepared by cutting the tonsils into small blocks of 2–3 mm and grinding the tissue through the sieve of a cell strainer (70 μm, BD Falcon, Bedford, MA, USA) with a syringe plunger. Cells were seeded in a 96 well plate at a concentration of 2 × 10^6^ cells per well. HLACs were cultured in RPMI 1640 supplemented with 15% (*v*/*v*) inactivated FCS, 2 mM l-glutamine, 100 U/mL penicillin and 100 μg/mL streptomycin, 2.5 μg/mL Fungizone, 1 mM sodium pyruvate, 1% (*v*/*v*) MEM non-essential amino acid solution and 50 μg/mL gentamicin.

### 2.3. Investigation of Gag Processing by Steady State Analyses

HeLa cells were transiently transfected with pNL*env*1 *wt*, or the Glu mutants thereof, carrying Glu to Ala mutations, either from the N- or from the C-terminus of p6. After over-night cultivation at 37 °C the cell culture supernatant was harvested and VLPs released into the supernatant were pelleted through 20% (*w*/*v*) sucrose by centrifugation (20,000× *g*, 4 °C, 90 min), washed with ice cold PBS, centrifuged (20,000× *g*, 4 °C, 90 min), and lysed in SDS-PAGE sample buffer containing 2% SDS. The cells were detached and washed twice in ice-cold PBS and lysed in RIPA buffer (see Section 2.1) supplemented with 1 mM PMSF, 5 mM NEM, and protease inhibitor cocktail cOmplete (Roche). The samples were analyzed by western blotting (see Section 2.6).

### 2.4. Time Course Analyses of VLP Release

HeLa cells were transiently transfected with pNL*env*1 *wt*, or the Glu mutants E6-29A or E0A. 24 h post transfection, cells were detached and washed twice in ice-cold PBS. After resuspension in RPMI 1640 medium, cells were aliquoted into 1.5 mL reaction tubes and incubated while gentle shaking at 37 °C for the indicated time periods. Cells were collected by centrifugation (3000× *g*, 4 °C) and lysed in RIPA buffer (see Section 2.1) supplemented with 1 mM PMSF, 5 mM NEM, and protease inhibitor cocktail cOmplete (Roche). VLPs released into the cell culture supernatant were pelleted through 20% (*w*/*v*) sucrose by centrifugation (20,000× *g*, 4 °C, 90 min), washed with 1 mL PBS, centrifuged (20,000× *g*, 4 °C, 90 min) and directly lysed in 2% SDS sample buffer. All samples were standardized for protein levels, analyzed by western blotting, stained with antibodies and processed in parallel as described below (see Section 2.6). To determine the quantity of VLPs released from the cells, the amount of CA in the VLP fraction was calculated relative to the total amount of Gag present intra- and extracellularly.

### 2.5. Detection of Ubiquitinated Gag

Detection of ubiquitinated Gag was carried out as described earlier [32,39]. Inactivation of the viral protease (PR^−^) was carried out by adding Nelfinavir to a final concentration of 10 μM upon transfection. HeLa cells were lysed with RIPA buffer containing protease inhibitors. Lysates were adjusted to 1% SDS (*w*/*v*) and denatured at 95 °C for 10 min, subsequently diluted 10 fold, and were then cleared by centrifugation at 20,000× *g* for 10 min. Gag content was recovered by immunoprecipitation with antibodies from HIV-1 patient sera pre-bound to GammaBind Plus Sepharose (GE Healthcare, Little Chalfont, UK).

### 2.6. SDS-PAGE and Western Blotting

Protein samples were separated by SDS-PAGE [40] and subsequently transferred onto PVDF membranes (GE Healthcare). Membranes were blocked with 3% bovine serum albumin and incubated with the appropriate primary antibody (Ab). Gag was detected by a rabbit Ab recognizing CA (Seramun, Heidesee, Germany). The mouse *anti*-transferrin receptor antibody was purchased from Life Technologies and the *anti*-β-actin antibody from Sigma-Aldrich (St. Louis, MO, USA). The *anti-*mouse and *anti-*rabbit secondary antibodies coupled to HRP were obtained from Dianova (Hamburg, Germany). Tagged proteins were detected by monoclonal antibodies (mAb) directly conjugated to horse radish peroxidase (HRP) specific for HA (Roche, Basel, Switzerland) or FLAG (Sigma-Aldrich, St. Louis, MO, USA). Protein bands were quantified using AIDA [41].

### 2.7. Expression Plasmids

The plasmid for expression of hemagglutinin (HA)-tagged ubiquitin (HA-Ub) was kindly provided by Hans-Georg Kräusslich [23]. The FLAG-ALIX expression construct is described elsewhere [42]. The pNL4-3 [43] derived expression constructs pNL*env*1 *wt* [44], the ∆PTAP mutant [29], or the G2A mutant [32] have been described previously. The codon optimized Gag expression plasmids, containing the SL sequence and harboring *wt* p6 or the exchange of the two PTAP motifs, have been described elsewhere [33,45]. All mutations concerning the Glu residues were introduced by site-directed mutagenesis (QuikChange^®^ Lightning; Agilent Technologies, Santa Clara, CA, USA) using complementary primers.

### 2.8. Flow Cytometry

For detection of H2-K^b^-bound SL-epitope, cells were stained with the allophycocyanin (APC)-conjugated 25D1.16 mAb (eBioscience, San Diego, CA, USA) diluted 1:100 in FACS buffer (5% (*v*/*v*) FCS, 0.02% (*v*/*v*) NaN_3_ in PBS). For intracellular Gag staining, cells were permeabilized using Cytofix/Cytoperm (BD Bioscience, San Jose, CA, USA). Gag was detected by staining with a FITC-labeled *anti-*CA mAb (KC57; Beckman Coulter, Brea, CA, USA) diluted 1:100 in Perm/Wash buffer (BD Bioscience). Flow cytometry was performed on a BD™ LSR II flow cytometer using BD FACSDiva™ software (BD Bioscience). Data were analyzed by using the FACS Express V3 software [46].

### 2.9. Single Round Infection Assay

HeLa TZM-bl indicator cells were seeded in 96-well plates in duplicates (4000 cells/well) and infected the next day with 1 ng VSV-G-pseudotyped virions standardized for p24 content by ELISA (Aalto Bio Reagents, Dublin, Ireland). After overnight incubation, fresh medium was added. 48 h later, cells were washed with PBS and infection was detected using the Gal-Screen chemiluminescent reporter gene assay system (Applied Biosystems, Waltham, MA, USA). β-Galactosidase activity was quantified as relative light units per second using a Synergy HT microplate reader and Gen5 Data Analysis Software (BioTek, Winooski, VT, USA).

### 2.10. Infection of HLA Cultures

For infection of HLA cultures (see Section 2.2), 2 × 10^6^ cells were infected overnight with virus preparations equivalent to 1 ng of p24, and cell culture supernatant was collected every second or third day post infection.

### 2.11. Determination of the Replication Capacity

Virus replication was assessed by quantification of the virus-associated reverse transcriptase activity by [^32^P]-TTP incorporation, using an oligo(dT)-poly(A) template as described elsewhere [47]. To determine the replication capacity of HIV-1 following infection with the indicated HIV-1_NL4-3_ variants, the respective replication profiles were depicted as diagram (*y*-axis: RT activity; *x*-axis: dpi).

### 2.12. Membrane Flotation

The protocol for membrane flotation was adapted from [39]. Briefly, HeLa cells were extensively washed and scraped in homogenization buffer (0.25 M sucrose, 1 mM EDTA, 20 mM Tris HCl pH 8.0) and lysed by sonication. The post-nuclear supernatant was adjusted to 40% iodixanol (OptiPrep; Axis Shield, Dundee, UK) and underlayed to 28% and 2.5% iodixanol. Samples were centrifuged at 109,000× *g* for 3 h. Seven fractions were collected from the top of the gradient and analyzed by western blot.

### 2.13. Peptide Synthesis

The stepwise solid-phase synthesis of the peptide amide was performed on an automated Odysse microwave peptide synthesizer from CEM on a 0.1 mM scale, using conventional Fmoc/tBu strategy (HBTU, DIEA, NMP). As solid support H-Gln(Trt)-HMPB-ChemMatrix resin (130 mg, loading 0.47 mmol/g) from pcas Biomatrix was used. The first 10 amino acids were coupled according to standard methodologies in an automated single coupling mode (50 °C, 300 s) all other 600 s. Fmoc deprotection was carried out with 0.1 M HOBt, 5% Piperazine in DMF (50 °C, 300 s). Cleavage of crude peptides from the resin was accomplished through treatment with 95% TFA, 2.5% water, 2.5% TIPS for 3.5 h. Preparative purification by high-pressure liquid chromatography (HPLC) was carried out on a Shimadzu LC-8A system with a VariTide RPC column (21.2 mm × 250 mm) and a water/acetonitrile system (Buffer A: 0.2% TFA in water, Buffer B: 0.2% TFA in water:acetonitrile, 1:4).

The purified peptides were dried by lyophilization and characterized by analytical HPLC and mass spec analysis.

### 2.14. NMR Spectroscopy

Prior to NMR analysis of p6E0A, the protein was dissolved in 600 μL 50% aqueous TFE-D_2_ and transferred to a Wilmad 528-PP-7 NMR tube. TFE-D_2_O was delivered by Cambridge Isotope Laboratories, Inc. (Tewksbury, MA, USA). The sample concentration was 2 mM. The 1D and 2D ^1^H NMR experiments (Total Correlation Spectroscopy (TOCSY), Nuclear Overhauser Effect Spectroscopy (NOESY) and Correlation Spectroscopy (COSY)) were performed at 600.13 MHz on a Bruker Avance 600 MHz instrument equipped with an UltraShield Plus magnet and a triple resonance cryoprobe with gradient unit [48]. The 2D NMR experiments were performed at 300 K without spinning with mixing times of 110 ms for the TOCSY experiments and 250 ms for the NOESY experiments, respectively.

## 3. Results

### 3.1. Mutation of the Glutamic Acids in p6 Impairs Virus Budding and Gag Processing

**Figure 1:**
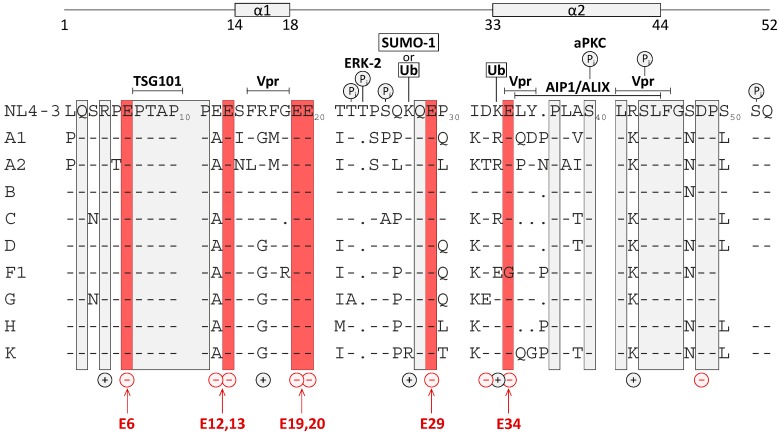
Amino acid sequence of p6 derived from the isolate HIV-1_NL4-3_ with previously identified structural and functional domains [50], charge distribution and sites of posttranslational modifications. Delineated in red are conserved Glu residues at positions 6, 13, 19, 20, 29, and 34. Positively and negatively charged residues, previously identified phosphorylation sites for ERK-2 and aPKC [35,36], attachment sites for ubiquitin [21] and SUMO-1 [22], binding domains for Tsg101 [8,9,10,34,51], ALIX [12], and Vpr [49] and structural domains identified by NMR [50] are indicated. Consensus sequences of p6 proteins derived from the M-group viruses [52] were aligned and conserved amino acids are boxed in grey.

Figure 1 shows the primary and secondary structure of p6 derived from the isolate HIV-1_NL4-3_ [43] as well as consensus sequences of p6 proteins derived from the M-group viruses. Despite its polymorphic character, p6 contains some highly conserved amino acid positions as depicted below. Intriguingly, all known functional sites within p6, such as the two previously described late assembly (l-) domains PTAP [27,28] and YP(X)_n_L [12,15] as well as the LXXLF motif, mediating Vpr incorporation into budding virions [49], overlap with these highly conserved regions. Containing 5 positive and 9 negative charges, p6 displays an unusually high charge density, which might also be controlled by phosphorylation. It has already been described that phosphorylation of p6 at position 40 by aPKC [36] and at residue Thr-23 by Erk-2 [35] plays a role in virus release and infectivity, although its biological function is still under debate [25,31].

Interestingly, most of the Glu residues within p6 are highly conserved among different M-group strains. However, the function of Glu residues with regard to virus assembly, budding, and Gag processing has not been investigated yet. For this reason, we conducted Ala scanning mutations with successive Glu to Ala exchanges in position 6, 12, 13, 19, 20, 29, and 34 of p6. These cumulative mutations, starting either from the N- or the C-terminus of p6, were introduced in the HIV-1 Gag expression plasmid pNL*env*1 [44]. The p6 mutant, completely devoid of Glu residues, was further on termed E0A. Serendipitously, the introduced Glu to Ala mutations did neither alter the amino acid sequence of the overlapping protein p6*, nor of the PR. For functional studies it was important that PR was not influenced at all, which was not the case as the overlapping *protease* gene starts at position 40 of the p6 ORF.

**Figure 2:**
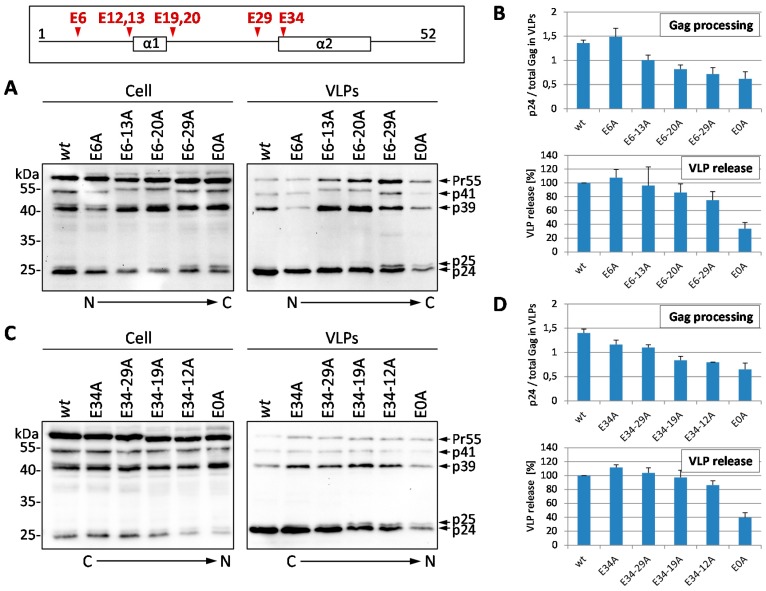
Successive mutation of glutamic acids in p6 leads to a dose-dependent defect in Gag processing and impaired VLP release. (**A**,**C**) HeLa cells were transiently transfected with the HIV-1 expression plasmid pNL*env*1, coding either for the *wt* or mutants carrying Glu to Ala mutations, either from the N- (**A**) or from the C-terminus (**C**) of p6. Whole cell lysates and VLP fractions were analyzed by western blotting using *anti*-CA antibodies (**B**,**D**). The VLP release was calculated as the amount of CA in the VLP fraction relative to the total amount of Gag detected in cell and VLP fractions. Values on the *y*-axis were adjusted to 100% for *wt*. Rate of Gag processing was calculated as the amount of CA relative to the total amount of Gag in each VLP fraction. Resulting ratios were normalized by mean of all values of each experiment (adapted from [53]). Values on the *y*-axis represent arbitrary units. Bars represent the mean values of 3 independent experiments ± SD.

**Figure 3:**
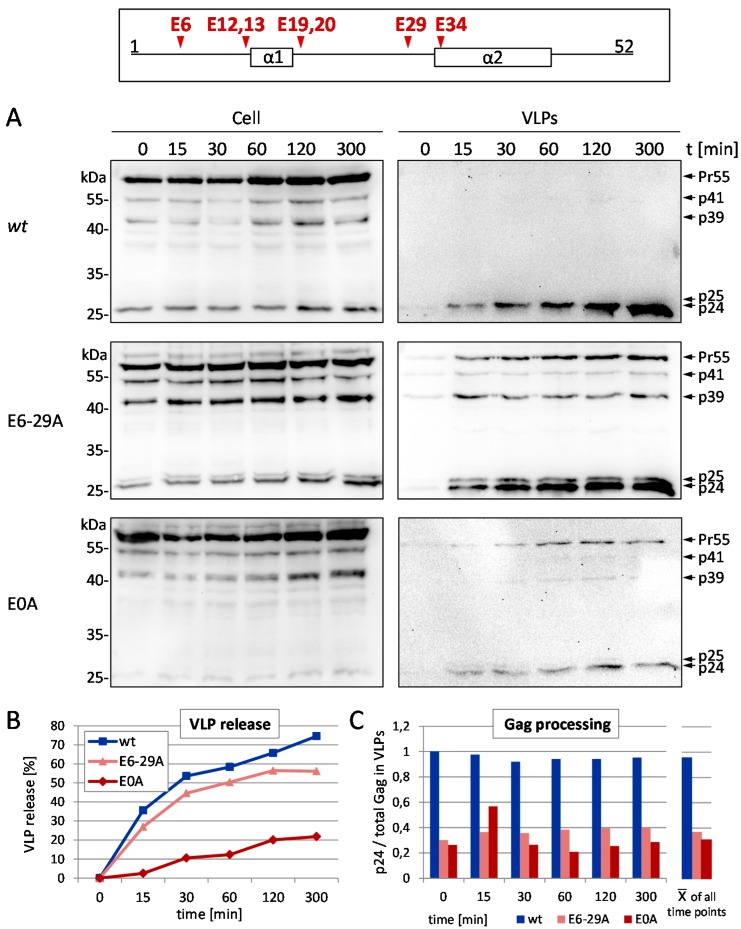
Mutation of all glutamic acids within p6 inhibits VLP release and Gag maturation. (**A**) HeLa cells were transiently transfected with pNL*env*1 *wt* or the Glu mutants E6-29A and E0A. VLP release was quantified by western blot time course analyses; (**B**) The percentage of VLPs released over time was calculated as the amount of CA detected in the VLP fraction *versus* the total amount of Gag recovered from cell and VLP fractions; (**C**) Gag processing was calculated as the amount of CA relative to the total amount of Gag in the VLP fraction. Values on the *y*-axis was adjusted to 1 for *wt* at *t* = 0.

Initially, HeLa cells were transiently transfected with mutants carrying successive Glu to Ala substitutions either from the N- to the C-terminus of p6, or the other way round, respectively. Western blot analyses of whole cell lysates and VLP fractions showed a dose-dependent (in terms of the number of Glu to Ala exchanges) defect in Gag processing with an accumulation of processing intermediates p41 (MA-CA-NC), p39 (MA-CA), and p25 (CA-SP1) (Figure 2A,C). This deficiency in Gag processing was most pronounced for the E0A mutant, which displayed a Gag processing defect of about 50% compared to that of the *wt* (Figure 2B,D). VLP release of the E6-13A mutant was reduced to approximately 95% that of the E6-20A mutant reaches about 85%, and the E6-29A mutant shows approximately 75% of the VLP release compared to the *wt*. A similar picture can be observed for the C- to N-terminal Glu mutants, where the E34-19A mutant shows about 95%, and the E34-12A mutant about 85% of *wt* VLP release (Figure 3A). Even though VLP release does decline in the same extent as the Gag processing defect upon mutation of the Glu residues, a maximal decrease in VLP release up to 40% of *wt* level was solely detected for the total E0A mutant. A stepwise effect of the intermediate mutants was only detectable for the accumulation of processing intermediates but hardly for the decline in virus release. (Figure 2B,D). The consecutive exchanges of the glutamic acids from either the N- or the C-terminus of p6 had similar effects on Gag processing and virus budding, indicating a position independent role of Glu residues in late functions of p6. Similar to other p6 mutants (∆PTAP, S40F [29,32,33]) the defect in Gag processing was particularly manifested by accumulation of the processing intermediate CA-SP1 (p25), indicating a delay in the final Gag processing step.

To further investigate the role of the Glu residues in budding and maturation, short term VLP release kinetics were conducted as described previously [33,54] and analyzed by western blot. The Glu mutant E6-29A, in which all glutamic acids are substituted, except for Glu-34, displayed a moderate decline in VLP release, while budding of the E0A mutant was clearly attenuated (Figure 3A). Although the efficiency of VLP release was only slightly affected as long as one Glu residue was present, Gag processing of the E6-29A mutant was significantly disturbed with pronounced accumulation of p25 (Figure 3A). Densitometric analyses of the western blot data illustrate that VLP release of the E6-29A mutant was only slightly reduced, by approximately 20%, whereas that of the E0A mutant was significantly diminished, by about 70% (Figure 3B). Consistent with data from steady state analyses (Figure 2), kinetic analyses revealed that both mutants exhibited reduced Gag processing, while only the E0A mutant showed maximal reduction in VLP release (Figure 3C).

### 3.2. Effect of Glu Mutants on Viral Infectivity and Replication Capacity

**Figure 4:**
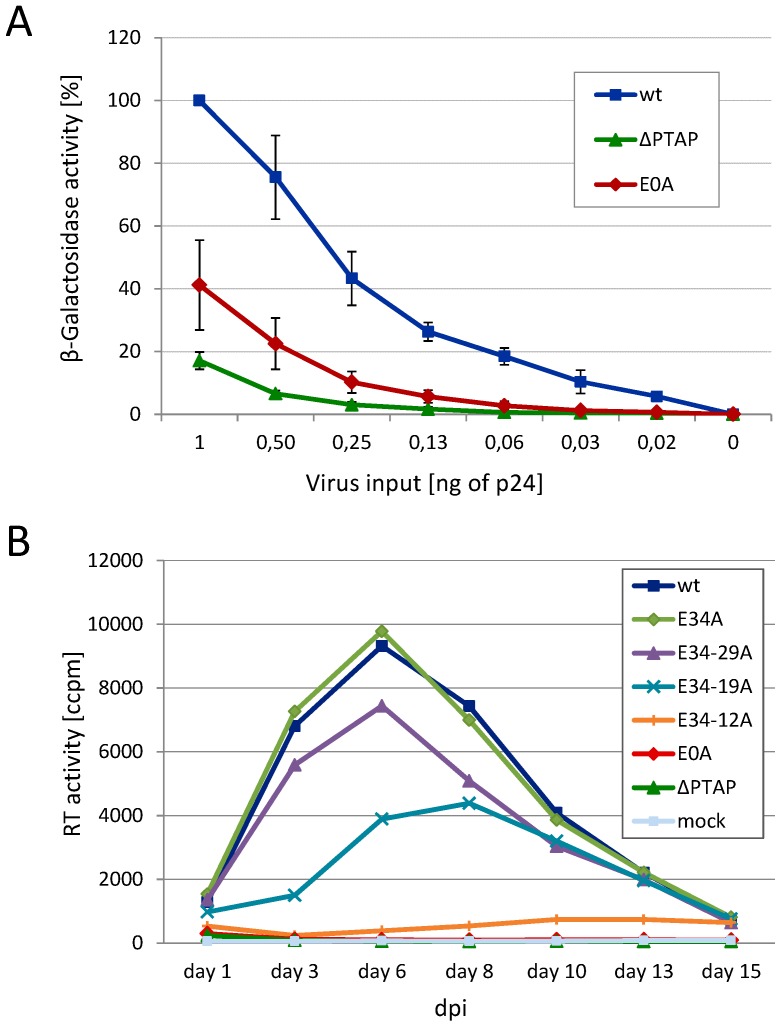
Mutation of glutamic acids reduces the infectivity and replication capacity of HIV-1. (**A**) TZM-bl cells were infected with VSV-G pseudotyped pNL*env*1 VLP stocks, standardized for p24. Infectious titers were determined by measuring the β-galactosidase activity. Bars represent the mean values of 3 independent experiments ± SD; (**B**) Infection of human lymphoid aggregate cultures (HLAC) with 1 ng virus standardized for p24 of HIV-1_NL4-3_ *wt* or the respective Glu mutants. Replication was assessed by quantification of the reverse transcriptase activity in cell culture supernatant collected every second or third day post infection (dpi) during 15 days of cultivation.

Having shown that the Glu residues seem to contribute to the function of p6 in late processes, we determined the influence of those Glu mutants on viral infectivity in single round infection assays. HeLa TZM-bl reporter cells were infected with VSV-G pseudotyped virions and infectivity was measured by β-galactosidase assay (Figure 4A). Consistent with data on Gag processing (Figure 2 and Figure 3) the E0A mutant exhibits reduced infectivity almost in the range of the ∆PTAP mutant. Since the infectivity rate was clearly diminished for the total Glu mutant E0A, it was legitimate to assume that the replication capacity of HIV-1 might also be affected by Glu mutations. Thus, HLACs (human lymphoid aggregate cultures), prepared from human tonsil tissue, were infected with HIV-1_NL4-3_ virus preparations standardized for p24, either *wt* or successive Glu to Ala mutants from the C- to the N-terminus. Measuring the RT activity revealed a stepwise decline of the replication capacity the more Glu residues were exchanged, resulting in complete loss of replication for the E0A mutant (Figure 4B), which was again comparable to the replication incompetent ∆PTAP mutant.

### 3.3. Budding Defect of the E0A Mutant Cannot Be Rescued by Overexpression of ALIX

**Figure 5:**
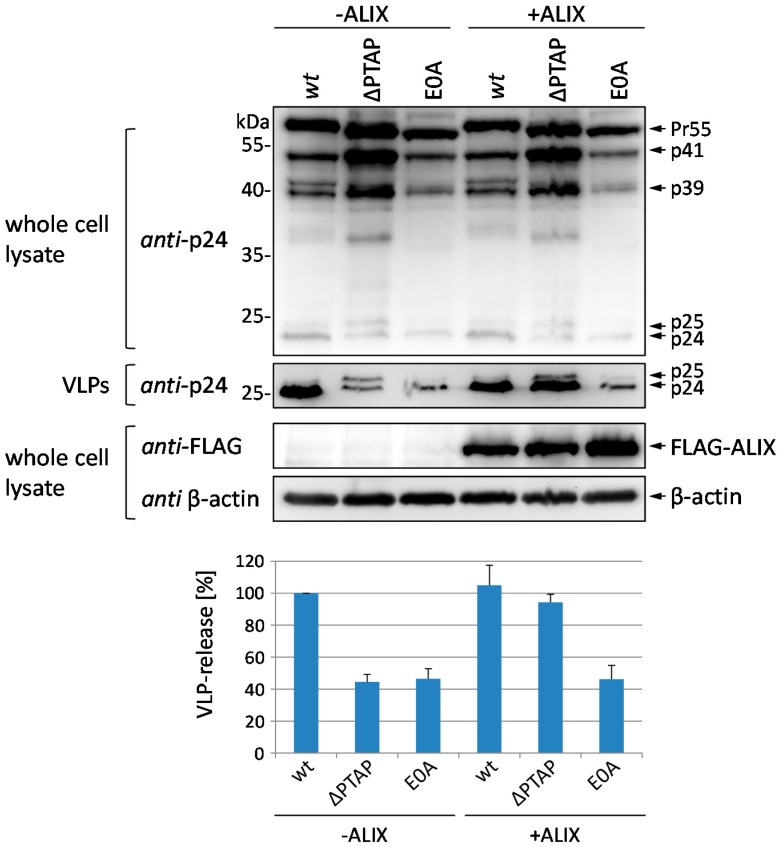
Defect in virus release of the E0A mutant cannot be rescued by ALIX overexpression. (**A**) HeLa cells were co-transfected with pNL*env*1 expression plasmids coding for *wt*, ∆PTAP or E0A and with FLAG-ALIX or empty control plasmids, respectively. VLP fractions and whole cell lysates were analyzed by western blot using an *anti*-CA. ALIX expression was detected using *anti*-FLAG antibodies; (**B**) VLP release was calculated as the amount of CA in the VLP fraction relative to the total amount of Gag detected in cells and VLPs. Bars represent the mean values of 3 independent experiments ± SD. Values on the *y*-axis were adjusted to 100% for *wt*.

The budding defect of ∆PTAP mutants can be reversed by overexpression of the cellular protein ALIX, which binds to the secondary YP(X)_n_L l-domain and interacts with the ESCRT complex [12,13,14]. Next, we investigated whether ALIX overexpression can also restore the defect in virus release observed for the E0A mutant. After coexpression of pNL*env*1 *wt*, the ∆PTAP or the E0A mutant with FLAG-tagged ALIX in HeLa cells, whole cell lysates and VLP fractions were analyzed by western blot stained with *anti*-CA antibodies. Calculation of the amount of CA in the VLP fraction relative to the total amount of Gag (Figure 5) revealed the typical budding defect of the ∆PTAP mutant that could be clearly rescued by ALIX overexpression. In contrast, ALIX had virtually no effect on the E0A mutant, neither in terms of Gag processing nor in terms of VLP release. To exemplify the delay in p25/24 processing for the E0A mutant, a long time exposure of western blot analysis of virus and cell fractions derived from *wt*, ∆PTAP, and E0A expressing HeLa cells is shown in Figure S1.

### 3.4. The Glu Residues in p6 Contribute to the Entry of Gag into the UPS

**Figure 6:**
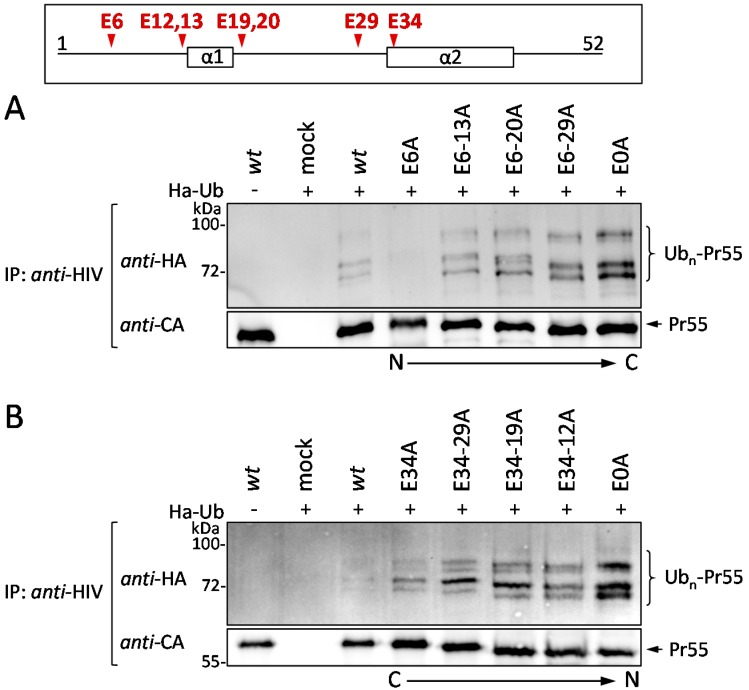
Successive mutation of the glutamic acids in p6 gradually increases HIV-1 Gag ubiquitination. (**A**,**B**) HeLa cells were co-transfected with HA-tagged ubiquitin and pNL*env*1 expression plasmids as indicated. Gag was immunoprecipitated from whole cell lysates using *anti*-HIV antibodies and Gag ubiquitination was detected by western blot stained for HA. The amount of precipitated Gag was detected by *anti*-CA staining.

According to previous studies, mutation of either PTAP or Ser-40 leads to increased polyubiquitination and enhanced MHC-I antigen presentation of Gag-derived epitopes [23,29,32,33,39,55]. For that reason, we wanted to know whether the Glu residues also affect the ubiquitination and thus the entry of Gag into the UPS. To address this question, HeLa cells were co-transfected with HA-tagged ubiquitin (HA-Ub) and pNL*env*1 *wt*, or the respective Glu mutants as indicated. After immunoprecipitation with Gag-specific antibodies, western blot analyses of precipitated Gag species were stained for HA (Figure 6A). Following co-expression of pNL*env*1 *wt* and HA-Ub, a specific pattern of high molecular weight species was observed, which most likely represents polyubiquitinated Gag. Upon gradual mutation of the Glu residues from the N- or from the C-terminus of p6, the intensity of the HA-Ub-signal was increased and reached its maximum level for the E0A mutant, irrespective of the direction of the consecutive Glu mutations (Figure 6A,B; lane 4–8).

It has already been well established that K48-linked polyubiquitination functions as a signal to direct proteins, and also HIV-1 Gag, into the proteasomal degradation pathway [56,57]. In the context of the HIV-1 p6 protein, mutations of the PTAP l-domain and Ser-40 have been demonstrated to cause elevated MHC-I presentation of Gag-derived epitopes as a result of an enhanced K48-linked polyubiquitination of Gag [32,33]. Since mutation of Glu residues increased the polyubiquitination of Gag (Figure 6), it was legitimate to investigate the MHC-I antigen presentation of Gag in this context. In our previous studies, we thoroughly demonstrated that the measurement of the presentation of Gag-derived epitopes on MHC-I molecules at the cell surface represents a highly sensitive and reliable approach to follow up the entry of Gag into the UPS [32,33,45,58].

As there are no antibodies available that efficiently recognize Gag-derived epitopes in the context of MHC-I, the ovalbumin-derived model epitope SIINFEKL (SL) was introduced into the p2 spacer region of Gag [45,59]. The pNL*env*1-SL expression plasmids, coding either for *wt* Gag or the respective Glu mutants, were then transfected into HeLa cells, which stably express the murine MHC-I allotype H2-K^b^ (HeLa-K^b^ [60]). Subsequently, antigen presentation was measured using the mAb 25D1.16, which specifically recognizes SL in complex with H2-K^b^ molecules [58].

**Figure 7:**
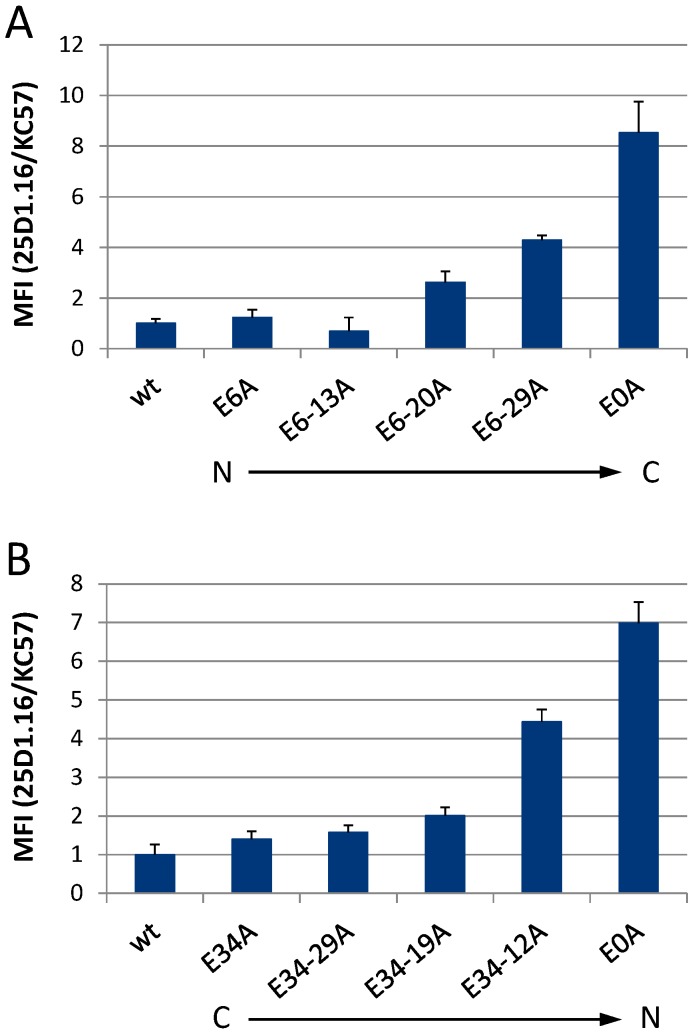
Successive mutation of the glutamic acids within p6 progressively increases the MHC-I antigen presentation of Gag-derived epitopes. HeLa-K^b^ cells were transiently transfected with pNL*env*1-SL expression plasmids coding for *wt* or the sequential Glu mutants of p6 from (**A**) the N- to the C-terminus or (**B**) the C- to the N-terminus, respectively. H2-K^b^-SL complexes presented on the surface of Gag-positive cells were quantified by flow cytometry using the mAb 25D1.16-APC [58]. After fixation and permeabilization, intracellular Gag was stained with *anti*-Gag Ab KC57-FITC. The mean fluorescence intensity (MFI) of the 25D1.16 staining, normalized to the MFI of the intracellular *anti*-Gag staining (see Figure S2) is shown. Bars represent mean values ± SD from three independent experiments.

Consistent with the data on Gag polyubiquitination (Figure 6), flow cytometry analyses revealed that progressive Glu mutation, either from the N- or from the C-terminus of p6, causes the presentation of increasing amounts of H2-K^b^-SL complexes at the cell surface (Figure 7) with the maximum reached for the total Glu deficient mutant E0A. Cells expressing E0A p6 displayed even an 8 times higher SL presentation, when compared to *wt* expressing cells. The MFI of intracellular Gag staining is shown in Figure S2. Altogether, these results demonstrate that Glu mutations enhance Gag polyubiquitination and entry of Gag into the MHC-I antigen presentation pathway.

### 3.5. Effect of Glu Mutations on the Structure of p6

Recently, we reported that the secondary structure of the p6 mutant S40F, which resembles that of the *wt* protein, seems to be present and functionally important as a determinant for membrane interaction of HIV-1 Gag [32]. The augmentation of membrane interaction of Pr55 Gag in case of the S40F mutant was caused by formation of a helix-dependent hydrophobic patch, consisting of Tyr-36 and Phe-40, which are close in space at the same surface of the C-terminal helix of p6 [32].

The E0A mutant, where all negatively charged Glu residues have been replaced with more hydrophobic Ala residues, is characterized by a reduced charge density compared with the *wt* protein. First, we wanted to investigate any potential effect of Glu residues on the secondary structure of p6. With exception of Glu-34, most Glu residues of *wt* p6 are not incorporated into regions with well-defined secondary structure. However, Glu-12, -13, -19, and -20 are located adjacent to the short N-terminal α-helix which is comprised by residues 14–18 [48,50]. Thus, it was essential to reveal if insertion of multiple Ala residues in the regions adjacent to the N-terminal α-helix of p6, the single mutation at position 29 in the flexible inter-helical region of the protein, and the Ala exchange at position 34 within the C-terminal α-helix could influence the extent and position of the secondary structure of p6.

**Figure 8:**
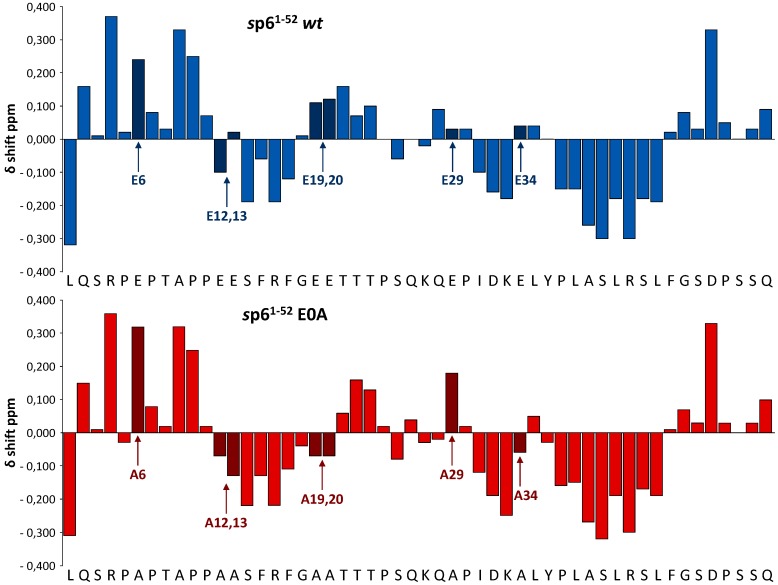
Mutation of Glu residues to Ala extends the N-terminal α-helix of p6. Chemical shift differences (ppm) of the α-protons between the experimental values and those for residues in a random coil for p6 *wt* [50] compared with the E0A mutant in 50% aqueous TFE-D_2_ at pH 3 at 300 K. All positive values for N-terminal residues adjacent to proline residues arise from an inherent effect of proline and not out of a structural perturbation, as described in ref. [62].

To investigate whether the multiple mutations of Glu residues of p6 had any effects on the position and extent of secondary structure of the protein at atomic resolution, NMR spectra of the E0A mutant dissolved in H_2_O-TFE-D_2_ (1:1) were obtained. It has been well established that α-proton chemical shifts greater than 0.1 ppm relative to the random coil values are qualitative indicators of protein secondary structure. A minimum of four adjacent residues with an upfield shift are indicative of an α-helix, whereas β-sheets require a minimum of three residues with downfield shifts [61]. After spectral assignment (Table S1), chemical shift index (CSI) plots were calculated (Figure 8) to indicate the location of the α-helical structure. In comparison to the *wt* protein mutation of Glu-12, 13, 19 and 20 to Ala led to an extension of the N-terminal α-helix of the E0A mutant which now includes residues 12–20 (Figure 8). On the other hand, mutation of Glu-29 and -34 to Ala did not influence the secondary structure of the protein (Figure 8). In summary, mutations of Glu to Ala, exemplified by the E0A mutant, cause enforcement of the α-helical structure of p6, particularly with extension of the N-terminal helix.

### 3.6. Mutation of Glu Residues Enhances Membrane Association of Gag

It was previously reported that enhanced membrane association of Gag is a prerequisite for its recognition by the cellular ubiquitination machinery, regardless of the presence of specific motifs [39]. Particularly, mutation of the PTAP l-domain has been shown to increase ubiquitination of Gag, dependent on its ability to bind to the plasma membrane [39,63]. Furthermore, it has been demonstrated that exchange of Ser-40 to Phe in p6 causes the formation of a hydrophobic patch which increases the membrane association and, as a consequence, enhances the polyubiquitination of Gag [32].

As we have already demonstrated that removing all Glu residues within p6 resulted in impaired virus release, Gag processing, infectivity and replication capacity, as well as in elevated Gag ubiquitination, and enhanced MHC-I antigen presentation, we investigated the impact of these mutations on membrane association of Pr55 by membrane flotation assays. HeLa cells were transiently transfected with the codon-optimized synthetic *gag* gene, *syngag*, which is expressed from a CMV promotor driven expression plasmid [64,65]. After whole cell lysis and density gradient centrifugation, 7 fractions were collected from the top of the gradient and the amount of Gag was determined by western blotting. As positive and negative controls, respectively, we used the myristoylation deficient MA G2A (∆myr) mutant, which displays a severely reduced membrane binding capacity [66,67], and the ∆PTAP mutant, that exhibits high levels of membrane-bound Gag [39]. To distinguish between membranous and cytosolic fractions, antibodies against β-actin and the membrane-bound transferrin receptor (TfR) were employed. Thereby, fractions 1 and 2 could be identified as membrane-containing, and fractions 5 to 7 as cytosolic layers.

**Figure 9:**
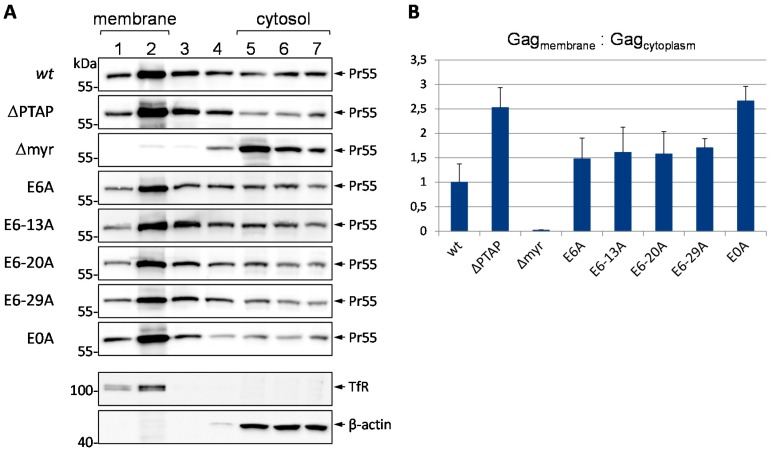
The mutations of Glu residues increase the membrane association of Pr55 Gag. (**A**) HeLa cells were transfected with *syngag* expression constructs coding for *wt* Gag, the p6 mutants ∆PTAP, E6A, E6-13A, E6-20A, E6-29A, E0A or the MA G2A mutant (∆myr). The cells were lysed by sonication and membrane flotation was performed. The fractions were collected and analyzed by western blot stained for CA. The western blots were reprobed with antibodies specific for β-actin and the transferrin receptor (TfR) to determine the cytosolic or membrane fractions, respectively. One representative example of the β-actin and TfR staining is shown; (**B**) The ratio of Pr55 Gag in the membrane fractions (1 and 2) *vs.* the cytosolic fractions (5 to 7) was calculated. Values on the *y*-axis were adjusted to 1 for *wt*. Bars represent the mean values of at least 3 independent experiments ± SD.

In line with the augmentation of both polyubiquitination and MHC-I antigen presentation, the E0A mutation increases the membrane association of Gag in comparison to the *wt* protein (Figure 9A). Densitometric analyses revealed that mutation of all Glu residues leads to an accumulation of Gag in the membranous fractions to the same extent as measured for the ∆PTAP mutant (Figure 9B). The p6 constructs with stepwise Glu mutations showed an intermediate phenotype in terms of membrane association regardless of number and respective positions of mutated Glu residues. This intermediate effect coincides with the observed effects of these mutants on Gag processing, ubiquitination and antigen presentation (Figure 2, Figure 6 and Figure 7).

Taken together, these results demonstrate that the augmentation of polyubiquitination observed for the E0A mutant is most likely due to its enhanced membrane association, occurring at a level comparable to that of the ∆PTAP mutant.

## 4. Discussion

The HIV-1 protein p6 has been demonstrated to be essential for budding of viral particles from the plasma membrane, a main function that is mediated by its primary l-domain PTAP. In contrast, mutation of the conserved Ser-40 residue to Phe was shown to increase the membrane association and polyubiquitination of Gag, and to cause aberrant HIV-1 core formation, reduced viral infectivity, and impaired CA-SP1 cleavage, whereas virus release was not affected [29,30,32]. Furthermore, mutation of residue Thr-23 of p6, which can be phosphorylated by the kinase Erk-2, was reported to result in an l-domain-like defect in virus release, aberrant core formation, and reduced infectivity [35], although a functional link to p6 phosphorylation could not be established in a later study [25]. Despite p6 being highly polymorphic, all of the established functional sites within p6 represent preserved regions of the protein.

In this study, we identified the conserved Glu residues as regulatory signals of p6 for membrane interaction of the Pr55 Gag polyprotein. Furthermore, the removal of these acidic residues causes enhanced polyubiquitination and subsequent MHC-I antigen presentation of Gag-derived epitopes. Most astonishingly, the phenotype induced by removal of all Glu residues in p6 seems to occur completely independent of the ESCRT machinery inasmuch as both l-domains remained intact in p6 E0A, and even overexpression of ALIX did not rescue the budding defect observed for the E0A mutant.

**Figure 10:**
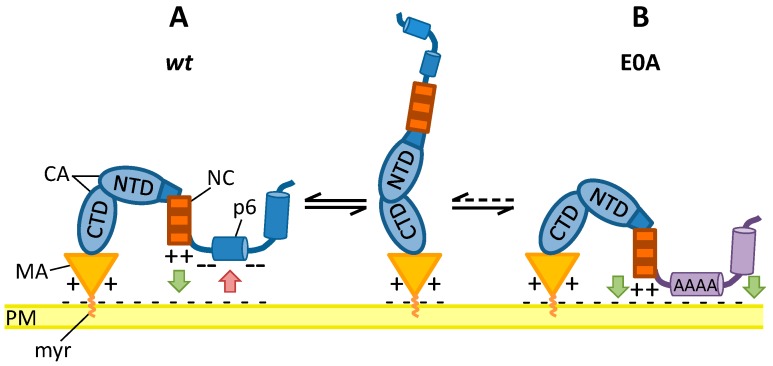
Hypothetical model of regulation of Gag membrane interaction. Green arrows indicate attraction, red arrows indicate repulsion. CTD: C-terminal domain; NTD: N-terminal domain; PM: plasma membrane.

It is commonly accepted that ubiquitin plays an essential role in retrovirus release but its mode of function has been controversially discussed [68,69,70]. Although some studies suggested that Gag ubiquitination contributes to efficient virus particle release via the ESCRT-complex [8,71,72], more recent studies indicate that ubiquitination of Gag can occur independently of the ESCRT complex and might be dispensable to drive viral particle release [32,33,63,73,74]. Jager *et al.* demonstrated that a late budding arrest causes enhanced ubiquitination of Gag by increasing its membrane association [39]. In concert with these results, we now show that the E0A mutant exhibits increased membrane binding of Gag, supporting the notion that membrane interaction of Gag is not only dependent on l-domain function (∆PTAP mutant [39]), but can also be affected by charge distribution (E0A mutant), or the introduction of a hydrophobic patch (S40F mutant [32]). In case of the S40F mutant, which in membrane flotation assays shows similarly increased proportions of Gag in the membrane fraction when compared to the ∆PTAP mutant [32], a direct membrane interaction was demonstrated by surface plasmon resonance (SPR) spectroscopy [48]. These observations led us to assume that in the *wt* situation the Glu residues within p6 might prevent prolonged binding of Gag to the cell membrane via charge repulsion between negatively charged membranous phospholipids and the negative charges of Glu residues in p6. This repulsion seems to be indispensable in order to ensure accurate progress of the late steps of virus replication. To illustrate this conception a preliminary, hypothetical model is depicted in Figure 10.

One possible explanation for the enhanced membrane association of the E0A mutant might be structural alterations induced by the non-conservative amino acid exchanges. To address this question, solution ^1^H NMR spectroscopy was conducted, and α-proton chemical shift differences revealed that mutations of the Glu residues leave the principal positions of the N- and C-terminal α-helices of p6 intact. Nevertheless, in case of the E0A mutant, the N-terminal helix of p6 is extended by residues 12, 13, 19, and 20, which most probably contributes to increased membrane binding of Gag via hydrophobic interactions. Although involvement of E34A in the C-terminal α-helix did not change its secondary structure, introduction of Ala might also increase the hydrophobicity of this helix and thus, in total, strengthen the membrane interaction, as observed by others for this C-terminal α-helix of p6, which appears to have an intrinsic propensity to interact with phospholipid bilayers [75].

Furthermore, it has been reported by others that the Pr55 Gag precursor undergoes a major conformational change during assembly [76,77,78]. In these studies, it was proposed that Gag was compact in solution with MA and NC in close proximity. Once MA anchors Gag to the plasma membrane, NC is also able to associate with the membrane via electrostatic interactions [77]. Upon binding of the viral RNA to NC and binding of Gag to the plasma membrane via MA, Gag is extended to its final conformation and this might be further stabilized by interprotein interactions [77]. Nevertheless, in these studies the authors used a Gag protein lacking the C-terminal p6 domain [76,77,78]. Membrane flotation assays using the complete Pr55 Gag protein now demonstrate enhanced membrane binding of Gag after mutation of Glu residues in p6. We propose a hypothetical model by which in the *wt* situation the Glu residues within p6 contribute to the extended shape of Gag through repulsion from the plasma membrane via their negative charges (Figure 10A). In consistency with this hypothesis is the finding that NC alone displays a strong interaction with the plasma membrane [79]. Ala substitution of all basic residues in NC significantly decreased membrane association of Gag [80]. Furthermore, it has been demonstrated that the p6 domain in the context of NCp15 folds back to the NC and interacts with the basic zinc fingers [81,82]. Conceivably, the nine negatively charged amino acids within p6, seven of which are constituted of Glu residues, are necessary to prevent prolonged binding of NC to the membrane via its basic residues, by counteracting its positive net charge.

Recently, it has been reported that duplication of the PTAP motif enhances the efficiency of NC-SP2-p6 processing in the context of PR mutations and under PI (protease inhibitor) treatment [83], also suggesting a functional interaction of NC and p6 during assembly and maturation. According to this, our data confirm that certain motifs in p6 contribute to efficient Gag processing, particularly the final CA-SP1 cleavage step.

Furthermore, the viral RNA could be identified as a negative regulator of Gag membrane binding [84,85]. The authors and several other studies showed that HIV-1 MA interacts with the viral RNA via its HBR (highly basic region) [86,87,88]. According to Chukkapalli *et al.*, this interaction negatively influences membrane binding of Gag [84]. Thus, one could speculate that the Glu residues within p6 contribute to the stretching of Gag upon binding of RNA to NC to finally enable efficient Gag processing.

Very recently, Wei *et al.* reported the interaction of Gag with a complex consisting of CCDC8, Obsl1, and the E3 ligase Cul7 at the plasma membrane, causing K48-linked polyubiquitination of Gag [63]. Accordingly, our results indicate that enhanced Gag membrane interaction of the E0A mutant contributes to increased polyubiquitination, which might occur via prolonged exposure to membrane resident E3 ligases like Cul7 during assembly. This finding is also consistent with the results obtained for the ∆PTAP and S40F mutants, which exhibit increased K48-linked polyubiquitination [23,32,33,55].

It is commonly known that K48-linked polyubiquitination designates proteins for degradation by the 26S proteasome [56]. Consistently, the E0A mutant preferentially enters the MHC-I pathway, as it shows a significantly enhanced presentation of Gag-derived epitopes even though the total amount of Gag seems not to be affected in steady state analyses. This result is in line with earlier studies, where an increase in MHC-I antigen presentation was not associated with a significant reduction of the half-life of Gag [33,45]. One possible reason for this phenomenon might be that only a small fraction of all newly synthesized Gag proteins becomes ubiquitinated and is subsequently directed into the UPS [57]. Moreover, reduction of the rate of Gag processing increases the amount of the Gag precursor, which is counteracted by proteasomal degradation.

Consistent with our previous studies, it has been shown by others that the efficiency of virus release was not affected by mutation of Ser-40 [29,30,31,32]. In contrast, the E0A mutant, which also increased Gag membrane binding, displayed a severe budding defect. These results indicate that the enhanced membrane association of Gag is somehow independent of virus budding, which led us to the hypothetical conclusion that the Glu residues in p6 fulfill two independent functions, namely the negative regulation of membrane binding of Gag as well as the maintenance of virus release. Remarkably, the latter process was only strongly diminished when all Glu residues were mutated, whereas the number of remaining Glu residues in p6 successively influences processes like ubiquitination, antigen presentation and Gag processing.

It has been shown previously that the budding defect observed after mutation of the PTAP motif can be rescued by ALIX overexpression [13]. As we observed that only the total Glu mutant E0A was completely impaired in virus release, it is legitimate to assume that the budding defect of the E0A mutant is not related to the well-established function of p6 as a docking site for the ESCRT system mediated by its two l-domains, inasmuch as both interaction sites for Tsg101 and ALIX remained vital in these experiments. Since both the ∆PTAP and the E0A mutant succumb to augmented polyubiquitination, but only the ∆PTAP mutant can be rescued by ALIX overexpression, one might conclude that the observed E0A phenotype occurs independently of l-domain interaction. In terms of the Glu residues, these results rather point towards a novel motif within p6 that besides the two l-domains, contributes to virus release. Nevertheless, one must also take into consideration that the E0A mutant, as it exhibits structural changes of the N-terminal helix, might have lost the ability to bind to Tsg101 and/or ALIX leading to the observed budding defect. This disability for the recruitment of critical ESCRT factors could also contribute to downstream events like increase of membrane association and polyubiquitination of Gag.

Moreover, it is conspicuous that p6 harbors a very high charge density. It creates the impression that the charges within p6 in general are necessary components of the protein as they are conserved among all HIV-1 subtypes aside from the amino acid conservation itself. For example in subtype F1 the Glu residue at position 34 is exchanged for Gly, which is compensated by an additional Glu residue at position 33 (Figure 1). Furthermore, the positively charged Arg-42 of the clone HIV-1_NL4-3_ is replaced by Lys in all other subtypes. In addition, at position 31, all subtypes display a positively charged Lys residue. Thus, the high charge density of p6 seems to be important for the functionality of the protein, to which the Glu residues might contribute. Aside from HIV-1, p6 related proteins of other retroviruses that appear to be involved in virus release also display a very high charge density, like p9 of the equine infectious anemia virus (EIAV), p6 of SIV and HIV-2, or p12 of murine leukemia virus (MuLV) [89,90,91].

Altogether, our results indicate that the duration of Gag at the cell membrane in the course of assembly and budding is regulated to be as short as necessary to allow the concerted action of virus release. Delay of Gag at the plasma membrane, caused by removal of the negative charges within p6, increases its access to E3 ligases such as Cul7 which was shown to disturb virus assembly and to direct Gag into proteasomal degradation [63].

In case of the E0A mutant, two concomitant mechanisms might contribute to the augmented membrane association. First, the loss of electrostatic repulsion between negatively charged residues in p6 and the phospholipid head groups of the cell membrane, and second, the extension of the N-terminal α-helix attended with the intensification of its hydrophobic character, that might interfere with efficient Gag assembly but might also negatively influence the interaction with the ESCRT components Tsg101 and/or ALIX leading to impaired virus release. However, one must also take into consideration that the charge density of p6 might not exclusively be influenced by the number of Glu residues, but might also depend on alterations in the status of p6 phosphorylation due to Glu mutations.

## 5. Conclusions

The cumulative data support a model by which p6 acts as negative regulator of Gag membrane interaction via its Glu residues, which shortens the duration of Gag at the plasma membrane necessary for efficient assembly and virus release.

## Supplementary Files

Supplementary File 1

## References

[1] Sundquist W.I., Krausslich H.G. (2012). HIV-1 assembly, budding, and maturation. Cold Spring Harb. Perspect. Med..

[2] Freed E.O. (2015). HIV-1 assembly, release and maturation. Nat. Rev. Microbiol..

[3] Wills J.W., Craven R.C. (1991). Form, function, and use of retroviral Gag proteins. AIDS.

[4] Gottlinger H.G., Sodroski J.G., Haseltine W.A. (1989). Role of capsid precursor processing and myristoylation in morphogenesis and infectivity of human immunodeficiency virus type 1. Proc. Natl. Acad. Sci. USA.

[5] Zhou W., Parent L.J., Wills J.W., Resh M.D. (1994). Identification of a membrane-binding domain within the amino-terminal region of human immunodeficiency virus type 1 Gag protein which interacts with acidic phospholipids. J. Virol..

[6] Darlix J.L., Godet J., Ivanyi-Nagy R., Fosse P., Mauffret O., Mely Y. (2011). Flexible nature and specific functions of the HIV-1 nucleocapsid protein. J. Mol. Biol..

[7] Lu K., Heng X., Summers M.F. (2011). Structural determinants and mechanism of HIV-1 genome packaging. J. Mol. Biol..

[8] Garrus J.E., von Schwedler U.K., Pornillos O.W., Morham S.G., Zavitz K.H., Wang H.E., Wettstein D.A., Stray K.M., Cote M., Rich R.L. (2001). Tsg101 and the vacuolar protein sorting pathway are essential for HIV-1 budding. Cell.

[9] VerPlank L., Bouamr F., LaGrassa T.J., Agresta B., Kikonyogo A., Leis J., Carter C.A. (2001). Tsg101, a homologue of ubiquitin-conjugating (E2) enzymes, binds the L domain in HIV type 1 Pr55(Gag). Proc. Natl. Acad. Sci. USA.

[10] Martin-Serrano J., Zang T., Bieniasz P.D. (2001). HIV-1 and Ebola virus encode small peptide motifs that recruit Tsg101 to sites of particle assembly to facilitate egress. Nat. Med..

[11] Demirov D.G., Ono A., Orenstein J.M., Freed E.O. (2002). Overexpression of the N-terminal domain of TSG101 inhibits HIV-1 budding by blocking late domain function. Proc. Natl. Acad. Sci. USA.

[12] Strack B., Calistri A., Craig S., Popova E., Gottlinger H.G. (2003). AIP1/ALIX is a binding partner for HIV-1 p6 and EIAV p9 functioning in virus budding. Cell.

[13] Usami Y., Popov S., Gottlinger H.G. (2007). Potent rescue of human immunodeficiency virus type 1 late domain mutants by ALIX/AIP1 depends on its CHMP4 binding site. J. Virol..

[14] Fisher R.D., Chung H.Y., Zhai Q., Robinson H., Sundquist W.I., Hill C.P. (2007). Structural and biochemical studies of ALIX/AIP1 and its role in retrovirus budding. Cell.

[15] Von Schwedler U.K., Stuchell M., Muller B., Ward D.M., Chung H.Y., Morita E., Wang H.E., Davis T., He G.P., Cimbora D.M. (2003). The protein network of HIV budding. Cell.

[16] McCullough J., Colf L.A., Sundquist W.I. (2013). Membrane fission reactions of the mammalian ESCRT pathway. Annu. Rev. Biochem..

[17] Caballe A., Martin-Serrano J. (2011). ESCRT machinery and cytokinesis: The road to daughter cell separation. Traffic.

[18] Carlton J.G., Caballe A., Agromayor M., Kloc M., Martin-Serrano J. (2012). ESCRT-III governs the Aurora B-mediated abscission checkpoint through CHMP4C. Science.

[19] Hanson P.I., Cashikar A. (2012). Multivesicular body morphogenesis. Annu. Rev. Cell Dev. Biol..

[20] Raiborg C., Stenmark H. (2009). The ESCRT machinery in endosomal sorting of ubiquitylated membrane proteins. Nature.

[21] Ott D.E., Coren L.V., Copeland T.D., Kane B.P., Johnson D.G., Sowder R.C., Yoshinaka Y., Oroszlan S., Arthur L.O., Henderson L.E. (1998). Ubiquitin is covalently attached to the p6Gag proteins of human immunodeficiency virus type 1 and simian immunodeficiency virus and to the p12Gag protein of Moloney murine leukemia virus. J. Virol..

[22] Gurer C., Berthoux L., Luban J. (2005). Covalent modification of human immunodeficiency virus type 1 p6 by SUMO-1. J. Virol..

[23] Gottwein E., Krausslich H.G. (2005). Analysis of human immunodeficiency virus type 1 Gag ubiquitination. J. Virol..

[24] Muller B., Patschinsky T., Krausslich H.G. (2002). The late-domain-containing protein p6 is the predominant phosphoprotein of human immunodeficiency virus type 1 particles. J. Virol..

[25] Radestock B., Morales I., Rahman S.A., Radau S., Glass B., Zahedi R.P., Muller B., Krausslich H.G. (2013). Comprehensive mutational analysis reveals p6Gag phosphorylation to be dispensable for HIV-1 morphogenesis and replication. J. Virol..

[26] Ott D.E., Coren L.V., Chertova E.N., Gagliardi T.D., Schubert U. (2000). Ubiquitination of HIV-1 and MuLV Gag. Virology.

[27] Huang M., Orenstein J.M., Martin M.A., Freed E.O. (1995). p6Gag is required for particle production from full-length human immunodeficiency virus type 1 molecular clones expressing protease. J. Virol..

[28] Gottlinger H.G., Dorfman T., Sodroski J.G., Haseltine W.A. (1991). Effect of mutations affecting the p6 gag protein on human immunodeficiency virus particle release. Proc. Natl. Acad. Sci. USA.

[29] Votteler J., Neumann L., Hahn S., Hahn F., Rauch P., Schmidt K., Studtrucker N., Solbak S.M., Fossen T., Henklein P. (2011). Highly conserved serine residue 40 in HIV-1 p6 regulates capsid processing and virus core assembly. Retrovirology.

[30] Watanabe S.M., Chen M.H., Khan M., Ehrlich L., Kemal K.S., Weiser B., Shi B., Chen C., Powell M., Anastos K. (2013). The S40 residue in HIV-1 Gag p6 impacts local and distal budding determinants, revealing additional late domain activities. Retrovirology.

[31] Radestock B., Burk R., Muller B., Krausslich H.G. (2014). Re-visiting the functional Relevance of the highly conserved Serine 40 Residue within HIV-1 p6(Gag). Retrovirology.

[32] Hahn F., Setz C., Friedrich M., Rauch P., Solbak S.M., Froystein N.A., Henklein P., Votteler J., Fossen T., Schubert U. (2014). Mutation of the highly conserved Ser-40 of the HIV-1 p6 gag protein to Phe causes the formation of a hydrophobic patch, enhances membrane association, and polyubiquitination of Gag. Viruses.

[33] Hahn S., Setz C., Wild J., Schubert U. (2011). The PTAP sequence within the p6 domain of human immunodeficiency virus type 1 Gag regulates its ubiquitination and MHC class I antigen presentation. J. Immunol..

[34] Demirov D.G., Orenstein J.M., Freed E.O. (2002). The late domain of human immunodeficiency virus type 1 p6 promotes virus release in a cell type-dependent manner. J. Virol..

[35] Hemonnot B., Cartier C., Gay B., Rebuffat S., Bardy M., Devaux C., Boyer V., Briant L. (2004). The host cell MAP kinase ERK-2 regulates viral assembly and release by phosphorylating the p6gag protein of HIV-1. J. Biol. Chem..

[36] Kudoh A., Takahama S., Sawasaki T., Ode H., Yokoyama M., Okayama A., Ishikawa A., Miyakawa K., Matsunaga S., Kimura H. (2014). The phosphorylation of HIV-1 Gag by atypical protein kinase C facilitates viral infectivity by promoting Vpr incorporation into virions. Retrovirology.

[37] Glushakova S., Baibakov B., Margolis L.B., Zimmerberg J. (1995). Infection of human tonsil histocultures: A model for HIV pathogenesis. Nat. Med..

[38] Glushakova S., Baibakov B., Zimmerberg J., Margolis L.B. (1997). Experimental HIV infection of human lymphoid tissue: Correlation of CD4^+^ T cell depletion and virus syncytium-inducing/non-syncytium-inducing phenotype in histocultures inoculated with laboratory strains and patient isolates of HIV type 1. AIDS Res. Hum. Retrovir..

[39] Jager S., Gottwein E., Krausslich H.G. (2007). Ubiquitination of human immunodeficiency virus type 1 Gag is highly dependent on Gag membrane association. J. Virol..

[40] Laemmli U.K. (1970). Cleavage of structural proteins during the assembly of the head of bacteriophage T4. Nature.

[41] (2008). AIDA.

[42] Votteler J., Iavnilovitch E., Fingrut O., Shemesh V., Taglicht D., Erez O., Sorgel S., Walther T., Bannert N., Schubert U. (2009). Exploring the functional interaction between POSH and ALIX and the relevance to HIV-1 release. BMC Biochem..

[43] Adachi A., Gendelman H.E., Koenig S., Folks T., Willey R., Rabson A., Martin M.A. (1986). Production of acquired immunodeficiency syndrome-associated retrovirus in human and nonhuman cells transfected with an infectious molecular clone. J. Virol..

[44] Khan M.A., Aberham C., Kao S., Akari H., Gorelick R., Bour S., Strebel K. (2001). Human immunodeficiency virus type 1 Vif protein is packaged into the nucleoprotein complex through an interaction with viral genomic RNA. J. Virol..

[45] Goldwich A., Hahn S.S., Schreiber S., Meier S., Kampgen E., Wagner R., Lutz M.B., Schubert U. (2008). Targeting HIV-1 Gag into the defective ribosomal product pathway enhances MHC class I antigen presentation and CD8^+^ T cell activation. J. Immunol..

[46] (2001). FCS Express.

[47] Willey R.L., Smith D.H., Lasky L.A., Theodore T.S., Earl P.L., Moss B., Capon D.J., Martin M.A. (1988). *In vitro* mutagenesis identifies a region within the envelope gene of the human immunodeficiency virus that is critical for infectivity. J. Virol..

[48] Solbak S.M., Reksten T.R., Hahn F., Wray V., Henklein P., Halskau O., Schubert U., Fossen T. (2013). HIV-1 p6—A structured to flexible multifunctional membrane-interacting protein. Biochim. Biophys. Acta.

[49] Kondo E., Gottlinger H.G. (1996). A conserved LXXLF sequence is the major determinant in p6gag required for the incorporation of human immunodeficiency virus type 1 Vpr. J. Virol..

[50] Fossen T., Wray V., Bruns K., Rachmat J., Henklein P., Tessmer U., Maczurek A., Klinger P., Schubert U. (2005). Solution structure of the human immunodeficiency virus type 1 p6 protein. J. Biol. Chem..

[51] Li L., Cohen S.N. (1996). Tsg101: A novel tumor susceptibility gene isolated by controlled homozygous functional knockout of allelic loci in mammalian cells. Cell.

[52] Los Alamos National Laboratory. http://www.hiv.lanl.gov.

[53] Degasperi A., Birtwistle M.R., Volinsky N., Rauch J., Kolch W., Kholodenko B.N. (2014). Evaluating strategies to normalise biological replicates of western blot data. PLoS ONE.

[54] Ott D.E., Coren L.V., Sowder R.C., Adams J., Schubert U. (2003). Retroviruses have differing requirements for proteasome function in the budding process. J. Virol..

[55] Martin-Serrano J., Perez-Caballero D., Bieniasz P.D. (2004). Context-dependent effects of L domains and ubiquitination on viral budding. J. Virol..

[56] Ciechanover A. (2005). Proteolysis: From the lysosome to ubiquitin and the proteasome. Nat. Rev. Mol. Cell Biol..

[57] Schubert U., Anton L.C., Gibbs J., Norbury C.C., Yewdell J.W., Bennink J.R. (2000). Rapid degradation of a large fraction of newly synthesized proteins by proteasomes. Nature.

[58] Porgador A., Yewdell J.W., Deng Y., Bennink J.R., Germain R.N. (1997). Localization, quantitation, and *in situ* detection of specific peptide-MHC class I complexes using a monoclonal antibody. Immunity.

[59] Rotzschke O., Falk K., Stevanovic S., Jung G., Walden P., Rammensee H.G. (1991). Exact prediction of a natural T cell epitope. Eur. J. Immunol..

[60] York I.A., Chang S.C., Saric T., Keys J.A., Favreau J.M., Goldberg A.L., Rock K.L. (2002). The ER aminopeptidase ERAP1 enhances or limits antigen presentation by trimming epitopes to 8–9 residues. Nat. Immunol..

[61] Wishart D.S., Sykes B.D., Richards F.M. (1992). The chemical shift index: A fast and simple method for the assignment of protein secondary structure through NMR spectroscopy. Biochemistry.

[62] Bruns K., Fossen T., Wray V., Henklein P., Tessmer U., Schubert U. (2003). Structural characterization of the HIV-1 Vpr N terminus: Evidence of *cis/trans*-proline isomerism. J. Biol. Chem..

[63] Wei M., Zhao X., Liu M., Huang Z., Xiao Y., Niu M., Shao Y., Kleiman L. (2015). Inhibition of HIV-1 assembly by coiled-coil domain containing protein 8 in human cells. Sci. Rep..

[64] Graf M., Bojak A., Deml L., Bieler K., Wolf H., Wagner R. (2000). Concerted action of multiple *cis*-acting sequences is required for Rev dependence of late human immunodeficiency virus type 1 gene expression. J. Virol..

[65] Deml L., Bojak A., Steck S., Graf M., Wild J., Schirmbeck R., Wolf H., Wagner R. (2001). Multiple effects of codon usage optimization on expression and immunogenicity of DNA candidate vaccines encoding the human immunodeficiency virus type 1 Gag protein. J. Virol..

[66] Freed E.O., Orenstein J.M., Buckler-White A.J., Martin M.A. (1994). Single amino acid changes in the human immunodeficiency virus type 1 matrix protein block virus particle production. J. Virol..

[67] Spearman P., Wang J.J., Vander Heyden N., Ratner L. (1994). Identification of human immunodeficiency virus type 1 Gag protein domains essential to membrane binding and particle assembly. J. Virol..

[68] Strack B., Calistri A., Accola M.A., Palu G., Gottlinger H.G. (2000). A role for ubiquitin ligase recruitment in retrovirus release. Proc. Natl. Acad. Sci. USA.

[69] Schubert U., Ott D.E., Chertova E.N., Welker R., Tessmer U., Princiotta M.F., Bennink J.R., Krausslich H.G., Yewdell J.W. (2000). Proteasome inhibition interferes with gag polyprotein processing, release, and maturation of HIV-1 and HIV-2. Proc. Natl. Acad. Sci. USA.

[70] Patnaik A., Chau V., Wills J.W. (2000). Ubiquitin is part of the retrovirus budding machinery. Proc. Natl. Acad. Sci. USA.

[71] Gottwein E., Jager S., Habermann A., Krausslich H.G. (2006). Cumulative mutations of ubiquitin acceptor sites in human immunodeficiency virus type 1 gag cause a late budding defect. J. Virol..

[72] Sette P., Nagashima K., Piper R.C., Bouamr F. (2013). Ubiquitin conjugation to Gag is essential for ESCRT-mediated HIV-1 budding. Retrovirology.

[73] Zhadina M., McClure M.O., Johnson M.C., Bieniasz P.D. (2007). Ubiquitin-dependent virus particle budding without viral protein ubiquitination. Proc. Natl. Acad. Sci. USA.

[74] Zhadina M., Bieniasz P.D. (2010). Functional interchangeability of late domains, late domain cofactors and ubiquitin in viral budding. PLoS Pathog..

[75] Salgado G.F., Vogel A., Marquant R., Feller S.E., Bouaziz S., Alves I.D. (2009). The role of membranes in the organization of HIV-1 Gag p6 and Vpr: p6 shows high affinity for membrane bilayers which substantially increases the interaction between p6 and Vpr. J. Med. Chem..

[76] Datta S.A., Curtis J.E., Ratcliff W., Clark P.K., Crist R.M., Lebowitz J., Krueger S., Rein A. (2007). Conformation of the HIV-1 Gag protein in solution. J. Mol. Biol..

[77] Datta S.A., Heinrich F., Raghunandan S., Krueger S., Curtis J.E., Rein A., Nanda H. (2011). HIV-1 Gag extension: Conformational changes require simultaneous interaction with membrane and nucleic acid. J. Mol. Biol..

[78] Munro J.B., Nath A., Farber M., Datta S.A., Rein A., Rhoades E., Mothes W. (2014). A conformational transition observed in single HIV-1 Gag molecules during *in vitro* assembly of virus-like particles. J. Virol..

[79] Kempf N., Postupalenko V., Bora S., Didier P., Arntz Y., de Rocquigny H., Mely Y. (2015). The HIV-1 nucleocapsid protein recruits negatively charged lipids to ensure its optimal binding to lipid membranes. J. Virol..

[80] Ko L.J., Yu F.H., Huang K.J., Wang C.T. (2015). HIV-1 matrix domain removal ameliorates virus assembly and processing defects incurred by positive nucleocapsid charge elimination. FEBS Openbio.

[81] Wu T., Gorelick R.J., Levin J.G. (2014). Selection of fully processed HIV-1 nucleocapsid protein is required for optimal nucleic acid chaperone activity in reverse transcription. Virus Res..

[82] Wang W., Naiyer N., Mitra M., Li J., Williams M.C., Rouzina I., Gorelick R.J., Wu Z., Musier-Forsyth K. (2014). Distinct nucleic acid interaction properties of HIV-1 nucleocapsid protein precursor NCp15 explain reduced viral infectivity. Nucleic Acids Res..

[83] Martins A.N., Waheed A.A., Ablan S.D., Huang W., Newton A., Petropoulos C.J., Brindeiro R.M., Freed E.O. (2015). Elucidation of the molecular mechanism driving duplication of the HIV-1 PTAP late domain. J. Virol..

[84] Chukkapalli V., Oh S.J., Ono A. (2010). Opposing mechanisms involving RNA and lipids regulate HIV-1 Gag membrane binding through the highly basic region of the matrix domain. Proc. Natl. Acad. Sci. USA.

[85] Alfadhli A., Still A., Barklis E. (2009). Analysis of human immunodeficiency virus type 1 matrix binding to membranes and nucleic acids. J. Virol..

[86] Purohit P., Dupont S., Stevenson M., Green M.R. (2001). Sequence-specific interaction between HIV-1 matrix protein and viral genomic RNA revealed by *in vitro* genetic selection. RNA.

[87] Cimarelli A., Luban J. (1999). Translation elongation factor 1-alpha interacts specifically with the human immunodeficiency virus type 1 Gag polyprotein. J. Virol..

[88] Hearps A.C., Wagstaff K.M., Piller S.C., Jans D.A. (2008). The N-terminal basic domain of the HIV-1 matrix protein does not contain a conventional nuclear localization sequence but is required for DNA binding and protein self-association. Biochemistry.

[89] Yuan B., Campbell S., Bacharach E., Rein A., Goff S.P. (2000). Infectivity of Moloney murine leukemia virus defective in late assembly events is restored by late assembly domains of other retroviruses. J. Virol..

[90] Yuan B., Li X., Goff S.P. (1999). Mutations altering the moloney murine leukemia virus p12 Gag protein affect virion production and early events of the virus life cycle. EMBO J..

[91] Zhai Q., Landesman M.B., Robinson H., Sundquist W.I., Hill C.P. (2011). Identification and structural characterization of the ALIX-binding late domains of simian immunodeficiency virus SIVmac239 and SIVagmTan-1. J. Virol..

