# Supplementary material for: Glutamic Acid Residues in HIV-1 p6 Regulate Virus Budding and Membrane Association of Gag
Source: Viruses. 2016 Apr 25;8(4):117. doi: 10.3390/v8040117 (PMC4848609; doi:10.3390/v8040117)
Supplement: Supplementary File 1 [file viruses-08-00117-s001.pdf]

Supplementary Materials: Glutamic Acid Residues in HIV-1 p6 Regulate Virus Budding and Membrane Association of Gag

Melanie Friedrich, Christian Setz, Friedrich Hahn, Alina Matthaei, Kirsten Fraedrich, Pia Rauch, Petra Henklein, Maximilian Traxdorf, Torgils Fossen and Ulrich Schubert

Table S1. ^1H chemical shifts of 2 mM p6 (1-52) E0A in TFE-H_2O (1:1; *v/v*) at 300 K.

	HN	Hα	Hβ	Hγ	Hδ	Hε	NH_2	Ar H
Leu-1		4.07	1.79	1.73	1.23/1.01			
Gln-2	8.66	4.52	2.18/2.08	2.44			7.50/6.72	
Ser-3	8.31	4.51	3.91/3.87					
Arg-4	8.10	4.74	1.93	1.79/1.73	3.25		7.19	
Pro-5		4.41						
**Ala-6**	8.03	4.67	1.40					
Pro-7		4.52	2.37/2.04	2.13	3.78/3.68			
Thr-8	7.82	4.37	4.25	1.25				
Ala-9	7.97	4.67	1.40					
Pro-10		4.69	2.40/1.96	2.12	3.89/3.61			
Pro-11		4.46	2.30/1.97	2.09	3.78/3.69			
**Ala-12**	8.03	4.28	1.43					
**Ala-13**	7.95	4.22	1.49					
Ser-14	7.92	4.28	3.92/3.86					
Phe-15	7.84	4.53	3.19/3.15					2/6 7.31
Arg-16	7.80	4.16	1.76	1.50	3.13		7.10	
Phe-17	7.94	4.55	3.26/3.08					2/6 7.28
Gly-18	8.10	3.99/3.87						
**Ala-19**	8.03	4.28	1.43					
**Ala-20**	8.00	4.28	1.49					
Thr-21	7.79	4.41	4.35	1.21				
Thr-22	7.80	4.51	4.32	1.27				
Thr-23	8.01	4.48	4.33	1.32				
Pro-24		4.46			3.91/3.78			
Ser-25	8.09	4.42	3.99/3.92					
Gln-26	7.98	4.41	2.18/2.06	2.42			7.36/6.65	
Lys-27	8.06	4.33	1.93/1.83	1.56/1.48	1.73	3.03	7.59	
Gln-28	8.25	4.35	2.22/2.14	2.45			7.40/6.69	
**Ala-29**	8.07	4.53	1.50					
Pro-30		4.46	2.33/2.02	2.11	3.83/3.72			
Ile-31	7.43	4.11	1.95	1.56/1.26/ 0.97/0.93				
Asp-32	8.09	4.57	2.95					
Lys-33	8.01	4.11	1.91	1.58/1.56	1.73	3.02	7.61	
**Ala-34**	7.77	4.29	1.51					
Leu-35	7.73	4.43	1.77	1.65	0.95/0.89			
Tyr-36	7.88	4.57	3.27/3.17					2/6 7.12 3/5 6.85
Pro-37		4.28	2.39/1.90	2.19/2.04	3.87			
Leu-38	7.23	4.23	1.82	1.73	1.01/0.94			

Table S1. *Cont.*

	HN	Hα	Hβ	Hγ	Hδ	Hε	NH_2	Ar H
Ala-39	8.27	4.08	1.47					
Ser-40	8.20	4.18	3.94/3.76					
Leu-41	7.88	4.19	1.92/1.83	1.71	0.98/0.93			
Arg-42	8.18	4.08	1.98/1.84	1.75	3.25/3.18		7.20	
Ser-43	7.95	4.33	4.06/3.96					
Leu-44	7.83	4.19	1.75	1.63/1.57	0.86/0.78			
Phe-45	8.03	4.67	3.36/3.04					2/6 7.33
Gly-46	8.04	4.04						
Ser-47	7.96	4.53	3.96					
Asp-48	8.28	5.09	3.09/2.88					
Pro-49		4.47	2.35	2.07	3.84/3.72			
Ser-50	8.00	4.50	4.00/3.93					
Ser-51	8.06	4.53	4.00/3.95					
Gln-52	8.05	4.47	2.27/2.06	2.40			7.39/6.62	

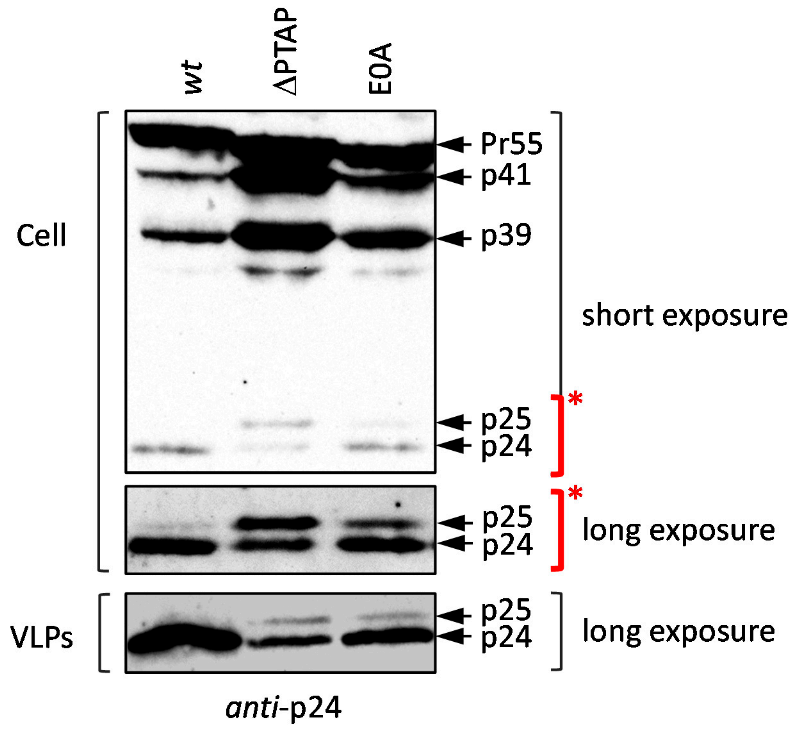

Figure S1. Western blot analysis of virus and cell fractions of *wt*, ΔPTAP and E0A expressing HeLa cells using a high performance chemiluminescence film. HeLa cells were transfected with pNL*env*1 expression plasmids directing the expression of *wt*, ΔPTAP, and E0A. VLP fractions and whole cell lysates were separated by SDS PAGE and analyzed by western blot using an anti-CA antibody. Short time exposure: 30 s; long time exposure: 5 min; * same region of the western blot.

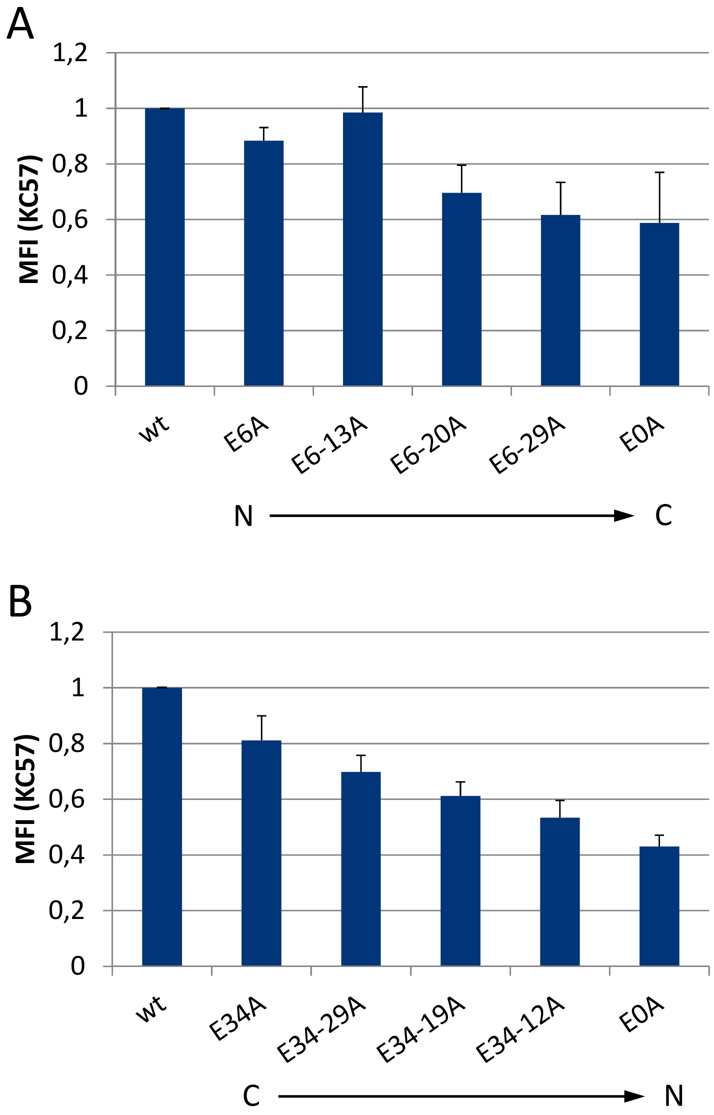

Figure S2. MFI of intracellular Gag staining corresponding to the experiments depicted in Figure 7. HeLa-K^b cells were transiently transfected with pNL*env*1-SL expression plasmids coding for *wt* or the sequential Glu mutants of p6 from (**A**) the N- to the C-terminus; or (**B**) the C- to the N-terminus, respectively. Intracellular Gag was stained with *anti*-Gag Ab KC57-FITC.

© 2016 by the authors; licensee MDPI, Basel, Switzerland. This article is an open access article distributed under the terms and conditions of the Creative Commons by Attribution (CC-BY) license (http://creativecommons.org/licenses/by/4.0/).
